# Unmet goals in the treatment of Acute Myocardial Infarction: Review

**DOI:** 10.12688/f1000research.10553.1

**Published:** 2017-07-27

**Authors:** Alejandro Farah, Alejandro Barbagelata

**Affiliations:** 1 Interventional Cardiology Department, San Bernardo Hospital, Salta, Argentina; 2 Universidad Católica de Buenos Aires, Buenos Aires, Argentina; 3 Department of Medicine, Duke University School of Medicine, Durham, NC, USA

**Keywords:** Myocardial Infarction, Reperfusion, Reperfusion Injury, Controlled reperfusion

## Abstract

Reperfusion therapy decreases myocardium damage during an acute coronary event and consequently mortality. However, there are unmet needs in the treatment of acute myocardial infarction, consequently mortality and heart failure continue to occur in about 10% and 20% of cases, respectively. Different strategies could improve reperfusion. These strategies, like generation of warning sign recognition and being initially assisted and transferred by an emergency service, could reduce the time to reperfusion. If the first electrocardiogram is performed en route, it can be transmitted and interpreted in a timely manner by a specialist at the receiving center, bypassing community hospitals without percutaneous coronary intervention capabilities. To administer thrombolytic therapy during transport to the catheterization laboratory could reduce time to reperfusion in cases with expected prolonged transport time to a percutaneous coronary intervention center or to a center without primary percutaneous coronary intervention capabilities with additional expected delay, known as pharmaco-invasive strategy. Myocardial reperfusion is known to produce damage and cell death, which defines the reperfusion injury. Lack of resolution of ST segment is used as a marker of reperfusion failure. In patients without ST segment resolution, mortality triples. It is important to note that, until recently, reperfusion injury and no-reflow were interpreted as a single entity and we should differentiate them as different entities; whereas no-reflow is the failure to obtain tissue flow, reperfusion injury is actually the damage produced by achieving flow. Therefore, treatment of no-reflow is obtained by tissue flow, whereas in reperfusion injury the treatment objective is protection of susceptible myocardium from reperfusion injury. Numerous trials for the treatment of reperfusion injury have been unsuccessful. Newer hypotheses such as “ *controlled reperfusion*”, in which the interventional cardiologist assumes not only the treatment of the culprit vessel but also the way to reperfuse the myocardium at risk, could reduce reperfusion injury.

## Introduction

**Figure 1.**
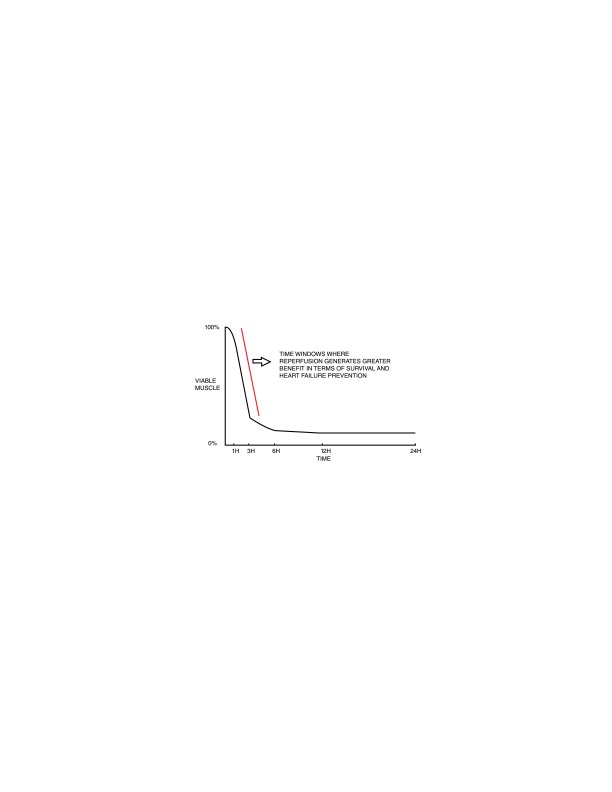
Relationship between time, extent of myocardial salvage, and mortality reduction.

**Figure 2.**
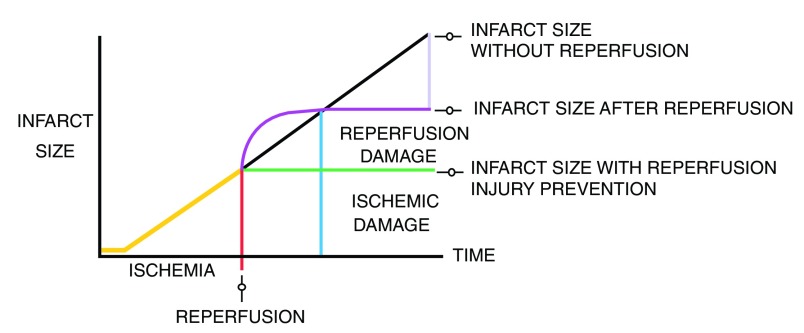
Ischemic injury and reperfusion injury contributions to final myocardial infarction size.

Atherosclerotic cardiovascular disease is the leading cause of death around the world ^1^. Acute myocardial infarction (AMI) is the event that causes most deaths or new cases of heart failure (HF) ^2– 5^. Early reperfusion therapy decreases the amount of myocardium damaged during an acute event and consequently mortality ^6, 7^. Primary percutaneous coronary intervention (PPCI) has become the optimal reperfusion strategy when performed in a timely manner ^8– 10^. However, there are unmet needs in the treatment of AMI, limiting the benefits that could be obtained with PPCI, since mortality and HF continue to occur in about 10% and 20% of cases each year, respectively ^2– 5^. In the current state of AMI treatment, two different stages can be recognized in which decrease of reperfusion benefits and in which the wavefront of necrosis could potentially be aborted. The first stage is the time from the onset of symptoms to reperfusion ( Figure 1). The second stage occurs during reperfusion ( Figure 2).

Efforts to optimize the benefit of PPCI are aimed at decreasing the time from onset of symptoms to reperfusion, reducing myocardial damage during the delay, and preventing reperfusion injury.

### Time reperfusion

The greatest benefit of reperfusion is obtained within the first 2 to 3 hours of ischemia ^11, 12^. The guidelines for the treatment of AMI indicate that the time from first contact with the health team for acquisition and interpretation of electrocardiogram (ECG) must be less than 10 minutes ^13, 14^. PPCI is chosen for reperfusion if it is done in a timely manner by a trained team within 120 minutes of the first medical contact (FMC) ^12, 15– 17^. If the FMC occurs in a PPCI center, the accepted delay to reperfusion is 90 minutes ^17, 18^ but preferably would be less than 60 minutes. Since most patients present to centers without PPCI capabilities, door-in to door-out time in the non-PPCI center has to be less than 30 minutes for patients transferred to a PPCI center ^12, 19, 20^.

If the FMC occurs in an institution without primary angioplasty (or in emergency medical services) and the expected delay for transfer for primary angioplasty has an estimated time of longer than 120 minutes, reperfusion with thrombolytic is recommended for patients without contraindications ^17, 21, 22^. In this case, the recommended time from arrival of the patient to starting the application of thrombolytic is less than 30 minutes ^17, 23, 24^.

But in the real world, the time from onset of symptoms to FMC varies widely, and usually patients wait 1.5 to 2 hours to seek medical attention, and only 66% of patients receive reperfusion within the recommendations of scientific guidelines ^25^. The variables related to delay from onset of symptoms to the FMC are the following: female gender; older age and those younger than 40 years; previous cardiovascular disease, particularly coronary heart disease; renal failure; and walk-in hospital presentation and geographical location ^26– 28^. The average time from onset of symptoms to FMC has not decreased in the last 10 years ^28, 29^. An additional delay is generated when the initial ECG is performed by a general practitioner who takes an average of 23.9 minutes ^30^. There is a close correlation between system delay and short- and long-term mortality; 1-hour delay in the system involves mortality of 15% at 3.4 years, and a delay of 3 hours increases mortality to 28.1% in the same period ^31^. Factors related to system delay are transfers from remote regions, presentation in a center not trained in reperfusion therapy, transfers between centers, delay for the administration of thrombolytics, and delayed activation of the catheterization laboratory.

**Figure 3.**
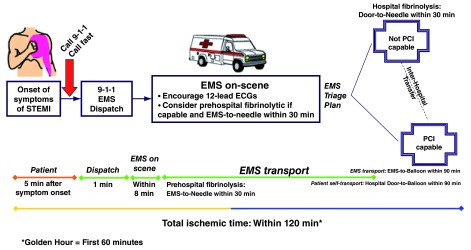
Strategies to optimize time to reperfusion. ECG, electrocardiogram; EMS, emergency medical services; PCI, percutaneous coronary intervention; STEMI, ST elevation myocardial infarction.

Strategies that could reduce the time to reperfusion are the following: education of the general population, generation of warning sign recognition ^32^ and being initially assisted and transferred by an emergency service; as in the case of cardiac arrest, they may benefit from receiving timely CPR ^33^. If the first ECG is performed during transport, it can be transmitted and interpreted by a specialist at the receiving center. This could allow the system to be activated while the patient is en route to the hospital ^34^. This might also allow thrombolytic therapy to be administered as a pharmaco-invasive strategy in those patients with a long transport time to the catheterization laboratory. The pharmaco-therapy with aspirin, clopidogrel, unfractionated heparin, and tenecteplase and subsequent interventionism demonstrated outcomes equivalent to those of primary angioplasty but with twice the major bleeding, so it has to be selected only in those patients with expected long delays for PPCI ^35^ and half the dose in the elderly population ( Figure 3).

### Reperfusion injury

**Figure 4.**
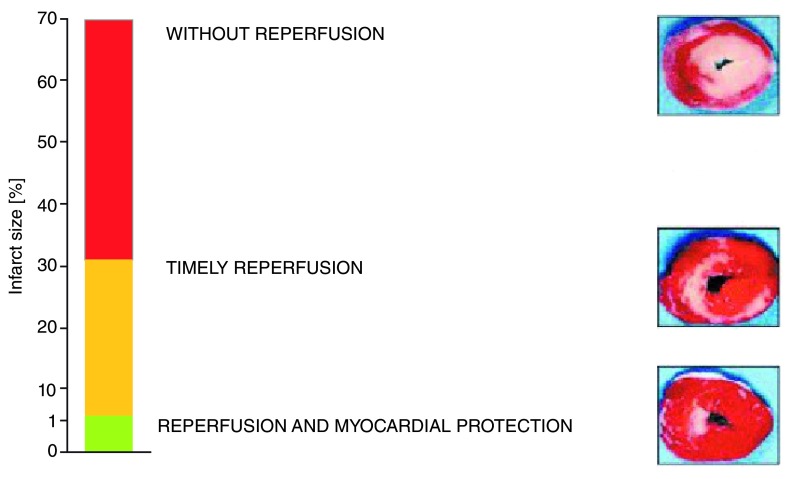
Potential benefits of reperfusion injury treatment.

Reperfusion therapy for AMI saves viable myocardium, but paradoxically the re-establishment of coronary blood flow also induces myocyte damage and death, limiting the full benefit of reperfusion in terms of reduction of infarct size and preservation of ventricular function ^36, 37^. Reperfusion itself can cause more damage and cell death; this process defines the phenomenon of reperfusion injury ^36, 38^ that potentially is prevented by applying additional therapies ^39^. Some evidence suggests that reperfusion injury may be responsible for up to 50% of the final myocardial damage during AMI ^36^ ( Figure 4).

**Figure 5.**
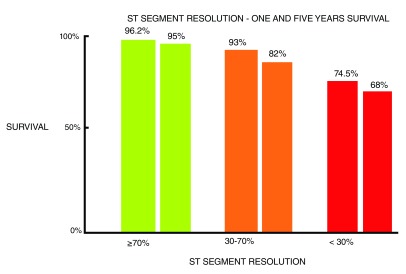
Relationship between lack of ST segment resolution and mortality.

The time from the symptom onset, diabetes, thrombolysis in myocardial infarction flow 0 in the baseline angiography, culprit lesion located at the proximal anterior descending artery, and presentation with HF are related to a higher chance of reperfusion injury ^40, 41^. Elevated white blood cells, increased platelet activation (size and reactivity), high thromboxane A2 and ET1 levels, hyperglycemia with or without diabetes, and C-reactive protein before reperfusion are predictors of this phenomenon ^42– 44^. It is possible that some degree of reperfusion injury is always present, but those patients with a short time from symptom onset or with previous angina seem less susceptible ^45, 46^. There is a useful rule of thumb to estimate its magnitude: the greater and more intense the ischemia, the greater the reperfusion injury ^41, 47– 49^. In everyday practice, the lack of ST segment resolution after achieving epicardial coronary flow is used as a marker of reperfusion failure. ST segment elevation does not decrease, mortality of AMI triples regardless of the achievement of adequate epicardial flow ^50, 51^ ( Figure 5).

***Diagnosis and differential diagnosis of reperfusion injury.*** The presence of reperfusion is a condition for reperfusion injury to exist. Clinical, electrocardiographic, and angiographic elements must be present. Clinical symptoms include increasing pain, anxiety, vegetative symptoms, and impaired hemodynamic status ^52, 53^. Electrocardiographic changes include ST segment elevation, onset of sinus tachycardia (by adrenergic discharge), malignant ventricular arrhythmias, extreme bradycardia, and electromechanical dissociation ^52– 54^. Angiographic elements include epicardial artery with signs of reperfusion and adequate antegrade flow and contrast extravasation in the microvasculature evidenced by persistent myocardial blush ^55– 57^.

**Figure 6.**
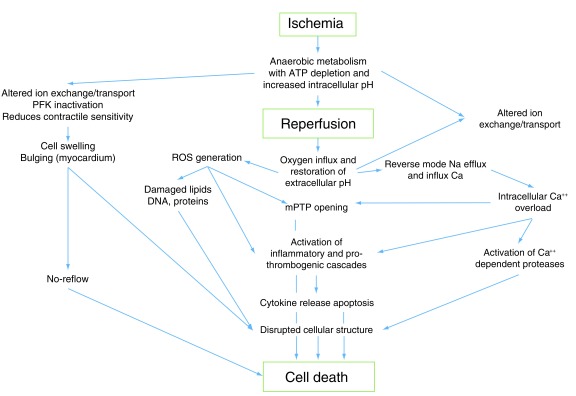
Physiopathologic events contributing to ischemic and reperfusion injury. PTP: membrane protein transition pore, ROS: reactive oxigen species, PFK: Phosphofructokinase.

Cell damage may be caused by different pathways during reperfusion ( Figure 6). The main event occurring during reperfusion and trigger of reperfusion injury is the abrupt increase of oxygen content in a medium with low pH (acidosis tissue caused by ischemia). In this scenario, the O _2_ reacts with hydrogen protons to reactive oxygen species (ROS), causing damage to DNA, protein, and lipid membranes, producing myocardial cell death ^58, 59^. In addition, ROS have pro-inflammatory effects, causing apoptosis and cell necroptosis ^60^. At the mitochondrial level, ROS open mitochondrial permeability transition pores, making them susceptible to irreversible damage ^60^. The damage produced by ROS at the level of the endoplasmic reticulum alters calcium dynamics, which in the context of acidotic reperfusion generates calcium influx into the sarcolemma, producing sustained hypercontraction and contraction band necrosis ^59– 61^. In addition, the influx of calcium-dependent proteases degrades structural components of the cell.

Reperfusion injury affects not only myocytes but also the microvasculature, where ROS produce direct damage of endothelial cells, causing increased permeability of the capillary wall and edema. ROS are chemotactic for neutrophils, activate complement, and trigger pro-thrombotic events ^60– 63^ ( Table 1 and Table 2). Finally, microvascular occlusion by perivascular edema, accumulation of neutrophils, and local thrombosis occur.

**Table 1.** Reperfusion injury: physiopathogenic elements.

Oxidative/nitrosative stress
Calcium overload
Endoplasmic reticulum stress
Mitochondrial dysfunction
Activation of apoptotic and autophagic pathways
Protein kinases
Epigenetic changes
Inflammation
Protein cleavage products and other degradation products

**Table 2.** Reactions that produce free radicals.

Superoxide production	O _2_ + e ^−^ : O _2_^−^
Hydrogen peroxide production	2H ^+^ O _2_ : H _2_O _2_
Haber-Weiss reaction	O _2_^−^ + H _2_O _2_ : O _2_ + 2OH
Fenton reaction	Fe ^2+^ + H _2_O _2_ : OH + OH ^−^ Fe ^3+^
Peroxynitrite production	O _2_^−^ + NO : ONOO
Peroxynitrous acid production	ONOO ^−^ + H ^+^ : ONOOH
Breakdown of peroxynitrous acid	ONOOH : OH _+_ NO _2_
NO _2_ and CO _3_ production	ONOO ^−^ + CO _2_ : NO _2_ + CO _3_

Reperfusion injury occurs by the influx of O _2_-saturated blood to a myocardial tissue that is made vulnerable by metabolic changes and a local internal environment that are produced during ongoing ischemia. Reperfusion injury is a rapid and irreversible phenomenon; therefore, the therapeutic strategy should focus on reducing the vulnerability of the myocardium or modify the blood that arrives to the susceptible muscle. Any therapy administered after reperfusion will be ineffective or of limited clinical benefit.

Different approaches were tested to reduce or prevent reperfusion injury and many of them failed ( Table 3) ^64^. Occasionally, conflicting results were found in selective therapies ^64^. Therefore, it is difficult to establish standardized treatment guidelines. Current scientific guidelines do not include reperfusion injury as a therapeutic target. It is important to note that, until recently, reperfusion injury and no-reflow were interpreted as a single entity ( Table 4) and we should differentiate them as different entities; whereas no-reflow is the failure to obtain tissue flow, reperfusion injury is actually the damage produced by achieving flow. Therefore, the way to treat no-reflow is to obtain tissue flow, whereas in reperfusion injury the treatment objective is to protect the susceptible myocardium from reperfusion injury. Another problem for the evaluation of clinical trials is that it is difficult to detect successful treatment for no-reflow and distinguish it from success in treating reperfusion injury if ultimately the common goal is to preserve the myocardium and there is no diagnosis of any of the phenomena before therapy is applied.

**Table 3.** Simplified evaluation scheme treatment of reperfusion injury.

Therapeutic target	Treatment	Route of administration	Result
Indeterminate	Hypothermia	IV	−
	Hypothermia	Peritoneal	−
MMTP	Delcasetrib	IV before reperfusion	−
	TR040303	IV before reperfusion	−
	Bendavia	IV before reperfusion	−
	Ciclosporin A	IV before reperfusion	+ −
Nitric oxide signaling	Nitrite sodium	IV	−
	Nitrite sodium	Intracoronary	−
	Nitric oxide	Inhaled	−
Pro-survival kinase	Copertide	IV	+
	Exenatide	IV	+
Indeterminate	Metoprolol	IV	+
Indeterminate	Post-conditioning	IC balloon inflations	+ + − −
Indeterminate	RIC	Limb ischemia	+ + + + + + + −

+, one trial with positive results; −, one trial with negative results; IC, intracoronary; IV, intravascular; MMTP, mitochondrial permeability transition pore; RIC, remote ischemic conditioning.

**Table 4.** Differential diagnosis with no-reflow.

	Reperfusion injury	No-reflow
Clinic	Sudden clinical deterioration	No changes to the state prior to reperfusion
Electrocardiography	ST segment elevation	ST unchanged with respect to electrocardiogram prior to reperfusion
Angiography	Persistent myocardial blush	No blush or slow blush
	Thrombolysis in myocardial infarction (TIMI) 2–3 at epicardial artery	TIMI 2–3 at epicardial artery

Given the pathophysiological difference of both entities, it may be considered that there is no reperfusion injury if no-reflow occurs. If a treatment is useful for no-reflow, this does not imply that it is useful for reperfusion injury. For example, perhaps thromboaspiration, glycoprotein IIb IIIa inhibitors, and vasodilators such as adenosine are effective for treatment of no-reflow but this does not mean that they avoid damage caused by ROS and pro-inflammatory cytokines. Likely, in a given patient, any therapeutic option for reperfusion injury is effective if the no-reflow phenomenon is solved first, the patient is being treated for an event that will not happen. Therefore the efficacy of treatment for each phenomena should be assessed separately in clinical trials. We also have to consider the treatment of both entities as predominantly preventive; therefore, clinicians need to start treatment before the phenomenon occurs and compare their effectiveness with controls.

It is reasonable to choose, as the definition of success for trials evaluating therapies in no-reflow, the presence of myocardial blush, whereas reperfusion injury therapies should define success by ST correction in the presence of positive myocardial blush ( Table 5).

**Table 5.** Theoretical model to evaluate success for no-reflow and reperfusion injury treatment.

Treatment	Myocardial Blush	ST	No-reflow/Reperfusion injury
	+	↓	Success/Success
	+	↑	Success/Failure
	−	↑	Failure/?
^a^	?	↑	?/Failure
^a^	?	↓	?/Success

^a^The existence of the latter two possibilities in this table is explained if one can evaluate a treatment for reperfusion injury by administering it by a microcatheter, balloon over the wire, or other similar device that can administer treatment before acting on the epicardial occlusion.

Pro-inflammatory and cytotoxic phenomena (not only local but systemic), which are triggered during ischemia and reperfusion, may continue to produce myocardial damage. These mechanisms could explain why some patients with successful reperfusion continue to lose myocardium (R wave of ECG) in the following reperfusion hours.

### Perspectives

The development of reperfusion therapies for AMI meaningfully reduced mortality. There are possibilities to optimize their use. Health teams should continue fighting to shorten the system delay and identify the best strategy according to the context in which they operate. To this end, initiatives such as Stent for Life are expanding around the world. There are working groups that conduct research in basic science, translational research, and clinical research against reperfusion injury, such as the Hatter Cardiology Institute, which (led by Derek Yellon) is making progress in myocardial protection using remote ischemic conditioning. We are working on primary controlled reperfusion and starting a clinical assay using intracoronary dextran plus vein blood through the balloon catheter before opening the artery. See Dextran Use for Primary Angioplasty Protection in Acute Myocardial Infarction. DUPAP Trial at ClinicalTrials.gov.

We hypothesized that developing treatment protocols for “ *continuous myocardial protection*” with different drugs, such as cyclosporine or other modulators of inflammation, administered from the time of diagnosis to the patient convalescence at the critical unit, could preserve myocardium during the delay of the system and during the early evolution of the event. To develop procedures of “ *controlled reperfusion*” where interventional cardiologists assume treatment not only for the culprit vessel infarction but also for myocardium could reduce reperfusion injury. The newer concept of “ *controlled reperfusion*” means deciding how to reperfuse (for example, post-conditioning with successive balloon inflations) and which adjunct compound to use during reperfusion (for example, administering to the ischemic myocardium, through dedicated catheters, prior to the opening of the artery, modified blood or enriched with drugs), preparing the myocardium for a more complete and definitive recovery. These two concepts—“ *continuous myocardial protection*” ** and “ *controlled reperfusion*”—open a wide field of research and development with potential benefits that could decrease myocardial damage and mortality.

## Abbreviations

AMI, acute myocardial infarction; ECG, electrocardiogram; FMC, first medical contact; HF, heart failure; PPCI, primary percutaneous coronary intervention; ROS, reactive oxygen species.
